# HLA-A24-restricted HTLV-I-specific CTL response reduces the HTLV-I proviral load but the HLA increases the risk of HAM/TSP

**DOI:** 10.1186/1742-4690-8-S1-A113

**Published:** 2011-06-06

**Authors:** Ryuji Kubota, Norihiro Takenouchi, Toshio Matsuzaki, Hiroshi Takashima, Shuji Izumo

**Affiliations:** 1 Center for Chronic Viral Diseases, Kagoshima University, Kagoshima 890-8544, Japan; 2 Department of Microbiology, Kansai Medical University, Moriguchi, Osaka 570-8506, Japan; 3 Department of Neurology and Geriatrics, Kagoshima University, Kagoshima 890-8544, Japan

## 

It is controversial whether HTLV-I-specific CTLs are beneficial or harmful to the host in the development of HAM/TSP. HLA-A2 reduces the risk of HAM/TSP and HLA-A2-restricted HTLV-I Tax11-19-specific CTL response reduces HTLV-I proviral load in asymptomatic HTLV-I carriers (ACs), suggesting that HLA-A2-restricted CTLs are beneficial to the host. Recently, HTLV-I Tax301-309 is newly identified as an immunodominant epitope restricted to HLA-A24 and frequency of Tax301-309-specific CTLs is high in HTLV-I-infected individuals. We investigated whether HLA-A24 also reduces the risk of HAM/TSP and compared the differences between HLA-A2- and HLA-A24-restricted Tax-specific CTL responses. We found that the allele frequency of HLA-A24 was significantly increased in HAM/TSP patients compared to ACs. The frequency of HTLV-I Tax301-309-specific CTLs was higher in HAM/TSP patients than that in ACs and negatively correlated with the HTLV-I proviral load in both HAM/TSP patients and ACs. In the comparison between HLA-A2/Tax11-19-specific CTLs and HLA-A24/Tax301-309-specific CTLs, the maximum responses by antigen stimulation were not different in IFN-gamma and MIP-1beta productions and CD107a expression, however, the functional avidity of the CTLs was 50-fold stronger in Tax301-309-specific CTLs than in Tax11-19-specific CTLs. This suggests that Tax301-309-specific CTLs more efficiently recognize HTLV-I-infected cells when the cells express low levels of viral proteins. Our data suggest that HLA-A24 increases the risk of HAM/TSP and that Tax301-309-specific CTLs may play a role in the pathogenesis of HAM/TSP even though they reduce the proviral load, or other factors related to HLA-A24 may affect the risk.

